# Epidemiological Trends and Hospitalization Profile of Alcohol-Related Mental and Behavioral Disorders in the State of São Paulo, the Most Populous State of Brazil, 2023

**DOI:** 10.1192/j.eurpsy.2025.1494

**Published:** 2025-08-26

**Authors:** J. R. Pinto Nasr, F. H. de Oliveira Bezerra, N. da Silva Fonseca, A. J. Bras Santos, T. Mosca Vidigal

**Affiliations:** 1 Universidade Paulista - Campus Campinas, Campinas, Brazil

## Abstract

**Introduction:**

Mental and behavioral disorders resulting from alcohol use are a significant public health issue. As the nation’s most populous state, São Paulo encounters distinct challenges in this domain. According to the 2022 Census, the most recent national demographic survey, the state of São Paulo’s population is estimated at 44,411,238, representing approximately 21.9% of Brazil’s total population of 203,080,756. Understanding the epidemiological profile of these disorders is crucial to better assess their impact on the healthcare system and to guide effective resource allocation and management strategies.

**Objectives:**

The present study aims to analyze statistical data related to hospitalizations due to mental and behavioral disorders caused by alcohol use in the state of São Paulo in 2023.

**Methods:**

A cross-sectional, descriptive, retrospective, and quantitative study was conducted, focusing on hospitalizations of individuals diagnosed with mental and behavioral disorders due to alcohol use in the state of São Paulo during 2023. Data were collected from the Department of Health Informatics of the Brazilian Unified Health System (DATASUS) within the “Hospital Information System of SUS” section, examining variables such as age range, gender, ethnicity, and length of hospital stay.

**Results:**

In 2023, São Paulo recorded 5,898 hospitalizations for alcohol-related mental and behavioral disorders, with total expenditures amounting to R$ 9,215,994.94. The average length of stay was 22.9 days, and the overall mortality rate was 0.64%, with 38 deaths. The highest number of hospitalizations occurred in the 35-39 years (12.1%), 40-44 years (14.7%), and 45-49 years (15.5%) age groups, which together accounted for 42.3% of all cases. Men represented 86.1% of hospitalizations, with an average length of stay of 23.4 days compared to 19.9 days for women. The ethnic distribution showed that 52.9% of hospitalizations were among White individuals, 8.3% were Black, 37.5% were Multiracial, and 0.7% were Asian. The longest average stays were among White individuals (25.1 days), followed by Black (22.2 days) and Multiracial (20.2 days). The overall mortality rate was 0.64%, with a slightly higher rate in men (0.65%) compared to women (0.61%). Black individuals had the highest mortality rate (1.23%), particularly among men (1.25%).

**Conclusions:**

This study underscores the public health impact of alcohol-related mental and behavioral disorders in the state of São Paulo, particularly among middle-aged men. Ethnic disparities in hospitalization duration and mortality suggest the need for targeted healthcare strategies to meet diverse demographic needs. These findings highlight the importance of tailored interventions and strategic resource allocation to reduce the burden of alcohol-related disorders and enhance health equity.

**Disclosure of Interest:**

None Declared

